# Supplementary material for: Observation of crystallisation dynamics by crystal-structure-sensitive room-temperature phosphorescence from Au(I) complexes
Source: Commun Chem. 2020 Oct 14;3:139. doi: 10.1038/s42004-020-00382-1 (PMC9814381; doi:10.1038/s42004-020-00382-1)
Supplement: Supplementary file 1 — Supplementary Information [file 42004_2020_382_MOESM1_ESM.pdf]

Supplementary Information for:

Observation of crystallisation dynamics by crystal-structure-sensitive room-temperature phosphorescence from Au(I) complexes

Yuki Kuroda, Masakazu Tamaru, Hitoya Nakasato, Kyosuke Nakamura, Manami Nakata, Kyohei Hisano, Kaori Fujisawa, and Osamu Tsutsumi*

Department of Applied Chemistry, Ritsumeikan University
1-1-1 Nojihigashi, Kusatsu 525-8577, Japan
Email: tsutsumi@sk.ritsumei.ac.jp

S2. Supplementary Methods

S2-1. Materials 2

S2-2. Preparation and characterisation of nanocrystals 5

S2-3. Photophysical properties 5

S2-4. Single-crystal X-ray structure analysis 5

S2-5. Powder X-ray diffraction analysis of nanocrystals 5

S2-6. Computational studies 6

S3. Supplementary Note 1

S3-1. Molecular structure and crystal packing structure 6

S3-2. Characterisation of micro- and nanocrystals 8

S3-3. Photophysical properties 8

S3-4. DFT calculation 11

S3-5. Effects of solvent on crystal structure and luminescence behaviour 14

S3-6. Mechanical-force effects on crystal structure and luminescence behaviour 17

S2. Supplementary Methods

S2-1. Materials.

The synthetic route for the preparation of trinuclear gold(I) complexes (DT*n*) is shown in Scheme 1.[1–4] Unless otherwise stated, all solvents and reagents were purchased from commercial suppliers and used without further purification. The complex, (tht)AuCl was synthesised according to a literature procedure.[1] ^{1}H NMR spectra were recorded on a JEOL ECS-400 spectrometer at 400 MHz using the residual proton in the NMR solvent as an internal reference. The complexes first synthesised in this study, DT4 and DT5, were fully characterised using high-resolution mass spectroscopy (HRMS), infrared spectroscopy (IR), and elemental analysis. Electrospray ionisation mass spectra (ESI-MS) were measured on a JMS-T1000LC (JEOL). IR spectra were recorded on a JASCO FT/IR-4100 spectrometer using a KBr pellet. The melting points of the final products were determined at the peak onset of differential scanning calorimetry (DSC) with heating and cooling rates of 1.0 °C/min. The synthetic details and characterisation of DT6 have been previously reported.[5]

Scheme S1. Synthetic route of DT*n*.

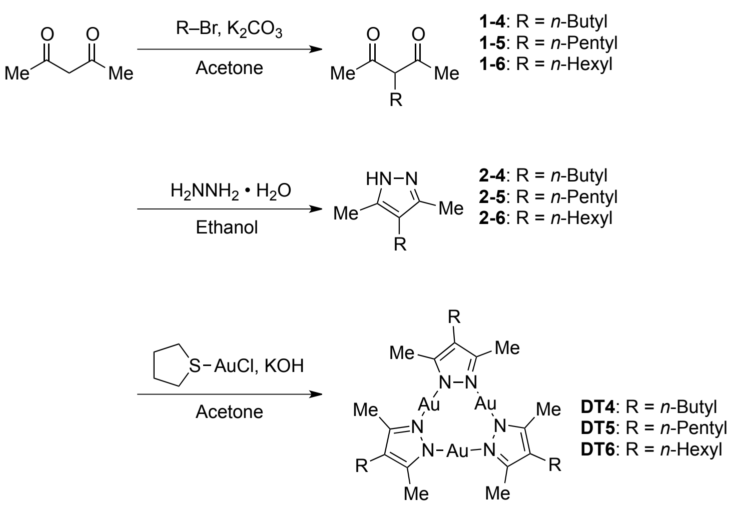

Synthesis of 1-n

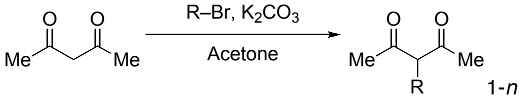

3-Butyl-2,4-pentanedione (1-4) Acetyl acetone (20 mL, 200 mmol), potassium carbonate (55 g, 400 mmol), and tetrabutylammonium bromide (6.4 g, 20 mmol) were added to 450 mL of acetone, and the mixture was refluxed. A solution of 1-bromobutane (2.8 mL, 20 mmol) in 50 mL of acetone was added, and the reaction mixture was refluxed for 18 h. The resultant solid was removed by filtration and the filtrate was concentrated by evaporation to give a yellow oil. The oil was poured into water and the product was extracted with ether. The organic layer was washed with brine and dried over anhydrous sodium sulfate. Following filtration and solvent removal, the crude product was purified by column chromatography on silica gel (*n*-hexane/ethyl acetate = 10/1) to give 0.73 g (4.7 mmol) of 1-4 as a colourless oil in 24% yield. ^{1}H NMR (400 MHz, $CDCl_3$, δ): 3.60 (t, J = 7.3 Hz; 1H; CH in pentanedione), 2.12 (s, 6H; CH_3 in pentanedione), 1.66–1.86 (br, 2H; $CH_2(CH_2)_2CH_3$), 1.29–1.38 (m, 4H; $CH_2(CH_2)_2CH_3$), 0.88 (t, J = 7.3 Hz; 3H; $CH_2(CH_2)_2CH_3$).

3-Pentyl-2,4-pentanedione (1-5) The title compound was prepared according to the above procedure as a colourless oil in 11 % yield. ^{1}H NMR (400 MHz, $CDCl_3$, δ): 3.60 (t, J = 7.3 Hz; 1H; 3-CH in pentanedione), 2.16 (s, 6H; 1,5-CH_3 in pentanedione), 1.81 (t, J = 7.5 Hz; 2H; 3-$CH_2(CH_2)_3CH_3$), 1.24–1.32 (m, 6H; 3-$CH_2(CH_2)_3CH_3$), 0.86 (t, J = 6.6 Hz; 3H; 3-$CH_2(CH_2)_3CH_3$).

Synthesis of 2-n

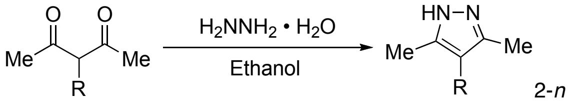

2-*n*

4-Butyl-3,5-dimethylpyrazole (2-4) 3-Butyl-2,4-pentanedione 1-4, (0.73 g, 4.7 mmol) was dissolved in 47 mL of ethanol. Hydrazine monohydrate (0.90 mL, 18 mmol) in 40 mL of ethanol was added to the solution with stirring. After the reaction mixture was refluxed for 16 h, the mixture was diluted with 40 mL of water. Ethanol was removed by evaporation, and the solution was acidified (*p*H = 1) with hydrochloric acid (10%). The product was extracted with dichloromethane. The organic layer was washed with brine and dried over anhydrous sodium sulfate. The solvent was removed completely under vacuum to give 0.53 g (3.5 mmol) of 2-4 as a white solid in 75% yield. ^{1}H NMR (400 MHz, $CDCl_3$, δ): 2.42 (s, 6H; CH_3 in pyrazole), 1.28–1.62 (m, 6H; $(CH_2)_3CH_3$), 0.92 (t, J = 7.3 Hz; 3H; $(CH_2)_3CH_3$).

4-Pentyl-3,5-dimethylpyrazole (2-5) The title compound was prepared according to the above procedure, in 39% yield. ^{1}H NMR (400 MHz, $CDCl_3$, δ): 2.39 (s, 6H; CH_3 in pyrazole), 1.21–1.46 (m, 8H; $(CH_2)_4CH_3$), 0.86 (t, J = 7.0 Hz; 3H; $(CH_2)_4CH_3$).

Synthesis of DTn

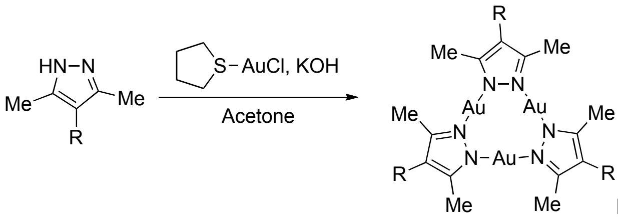

DT*n*

Tris(μ-3,5-dimethyl-4-butylpyrazolato-*N,N*)trigold(I) (DT4). 4-Butyl-3,5-dimethylpyrazole, 2-4 (0.53 g, 3.5 mmol) was dissolved in 150 mL of acetone, and potassium hydroxide in methanol (1.0 mol/L, 2 mL) was added to the solution. (tht)AuCl (0.58 g, 1.8 mmol) dissolved in 50 mL of acetone was added dropwise to the reaction mixture, with stirring. After the solution was stirred at room temperature for 17 h, the resultant precipitate was collected by filtration. The crude product was purified on a silica gel column (eluent: CH_2Cl_2) and then recrystallised from a mixture of dichloromethane and acetone to give 49 mg (47 μmol) of DT4 as colourless needles in 26% yield. mp 237 °C. ^{1}H NMR (400 MHz, $CDCl_3$, δ): 2.34 (t, J = 7.9 Hz; 6H; $CH_2(CH_2)_2CH_3$), 2.20 (s, 18H; CH_3 in pyrazole), 1.31–1.43 (m, 12H; $CH_2(CH_2)_2CH_3$), 0.91 (t, J = 7.3 Hz; 9H; $CH_2(CH_2)_2CH_3$). FTIR (KBr, cm^{-1}): 2954, 2919, 2852, 2362, 1637, 1516, 1456, 1372, 1225, 775, 726, 681. HRMS-ESI (*m/z*): $[M + Na]^+$ calcd for $C_{27}H_{45}Au_3N_6Na$, 1067.25999; found, 1067.26007. Anal calcd for $C_{27}H_{45}Au_3N_6$: C, 31.04; H, 4.34; N, 8.05; Au, 56.57. Found: C, 30.81; H, 4.13; N, 8.01; Ash, 52.4.

Tris(μ-3,5-dimethyl-4-pentylpyrazolato-*N,N*)trigold(I) (DT5). The title compound was prepared according to the above procedure, in 69% yield. mp 174 °C. ^{1}H NMR (400 MHz, $CDCl_3$, δ): 2.32 (t, J = 7.5 Hz; 6H; $CH_2(CH_2)_3CH_3$), 2.17 (s, 18H; CH_3 in pyrazole), 1.53–1.26 (m, 18H; $CH_2(CH_2)_3CH_3$), 0.89 (t, J = 7.3 Hz; 9H; $CH_2(CH_2)_3CH_3$). FTIR (KBr, cm^{-1}): 2955, 2923, 2853, 2358, 1638, 1515, 1453, 1372, 1224, 770, 725, 680. HRMS-ESI (*m/z*): $[M + Na]^+$ calcd for $C_{30}H_{51}Au_3N_6Na$, 1109.30694; found, 1109.30691. Anal calcd for $C_{30}H_{51}Au_3N_6$: C, 33.16; H, 4.73; N, 7.73; Au, 54.38. Found: C, 32.88; H, 4.43; N, 7.75; Ash, 54.28.

tris(μ-3,5-dimethyl-4-hexylpyrazolato-*N,N*)trigold(I) (DT6). The title compound was obtained according to a previously reported synthetic procedure, in 65% yield.[5] mp 134 °C. ^{1}H NMR (400 MHz, $CDCl_3$, δ): 2.31 (t, J = 7.5 Hz; 6H; $CH_2(CH_2)_4CH_3$), 2.14 (s, 18H; CH_3 in pyrazole), 1.28–1.41 (m, 24H; $CH_2(CH_2)_4CH_3$), 0.88 (t, J = 6.8 Hz; 9H; $CH_2(CH_2)_4CH_3$). FTIR (KBr, cm^{-1}): 2954, 2922, 2852, 2360, 1638, 1515, 1431, 1374, 1228, 772, 721, 685. HRMS-ESI (*m/z*): $[M + Na]^+$ calcd for $C_{33}H_{58}Au_3N_6$, 1129.37195; found, 1129.37256. Anal calcd for $C_{33}H_{57}Au_3N_6$: C, 35.11; H, 5.09; N, 7.45; Au, 52.35. Found: C, 34.88; H, 4.94; N, 7.37; Ash, 48.4.

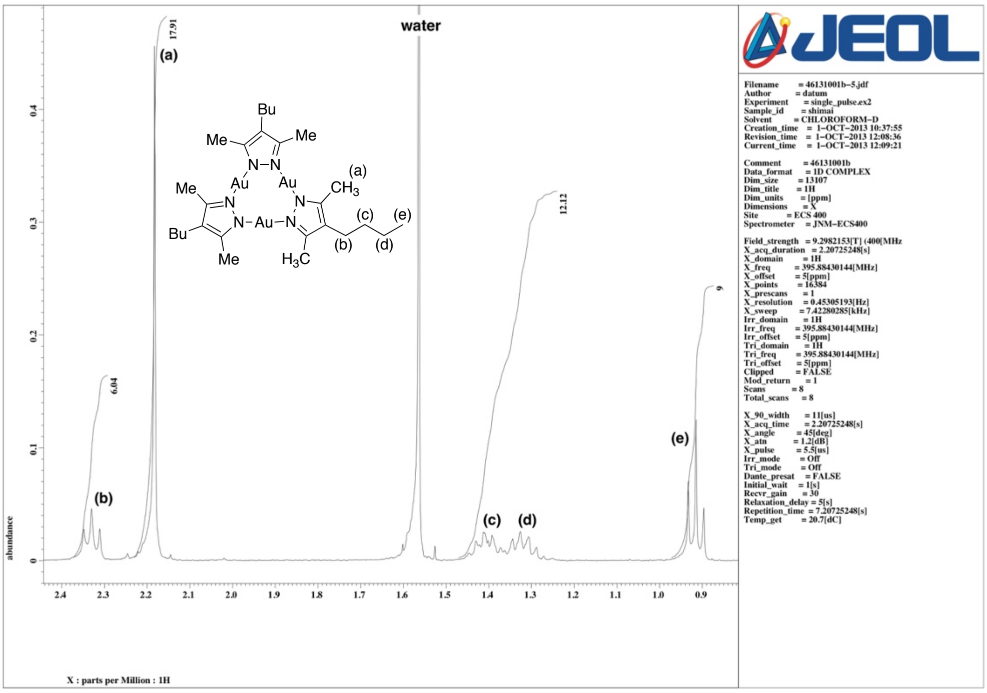

Supplementary Figure 1. ^{1}H NMR spectrum of DT4 in $CDCl_3$.

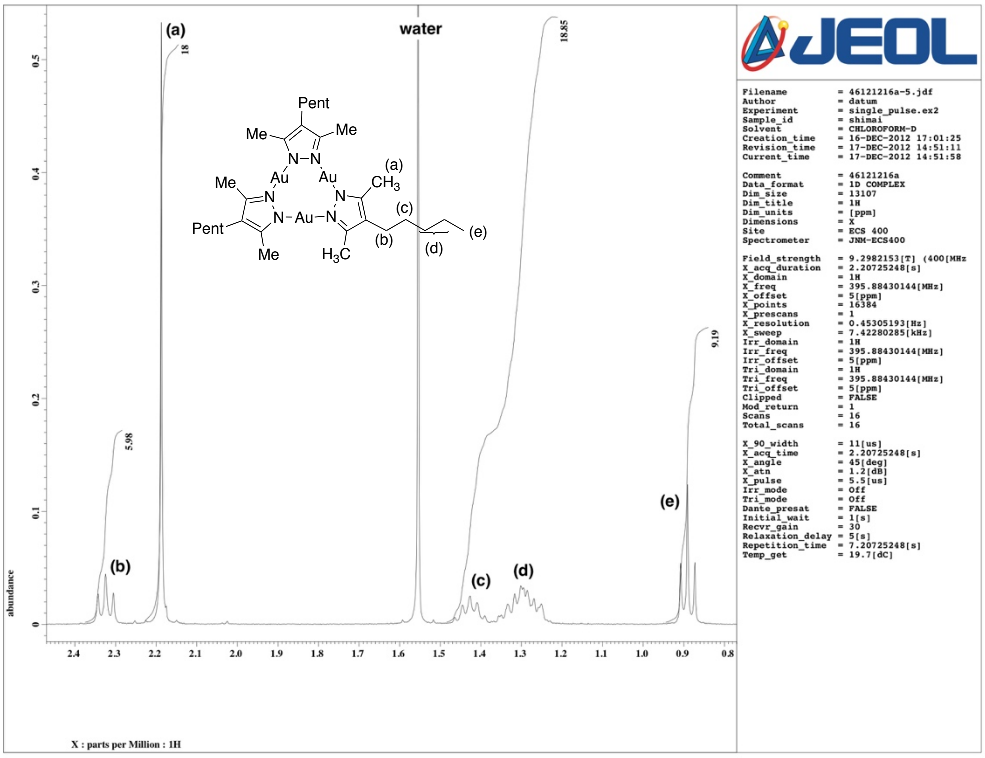

Supplementary Figure 2. ^{1}H NMR spectrum of DT5 in $CDCl_3$.

S2-2. Preparation and characterisation of nanocrystals

Preparation of nano- and microcrystals by reprecipitation

A THF solution of DT*n* (5.0 mmol L^{-1}, 50 μL) was added to 10 mL of deionised water under vigorous magnetic stirring at 1400 rpm at room temperature to obtain DT*n* nanocrystals. The resultant nanocrystals were collected by filtration of the suspension with a filter (pore size, 0.1 μm; 025010MFPES, ASONE).

Using the same method with a $CHCl_3$/methanol solvent system, crystals with a diameter of ~1 μm were obtained just after reprecipitation.

Preparation of nano- and microcrystals by mechanical milling

The nanocrystals were prepared by ball milling as well, using a PULVERISETTE 7 Planetary Ball Mill (FRITSCH, Germany). Bulk crystals of DT*n* (20 mg), methanol, and water (2/5 vol/vol, 1.2 mL) mixed solvent, and zirconia balls (ϕ = 1.0 mm, 303 mg) were placed in a glass vial (1.5-mL volume). The vial was sealed and placed into a stainless pot, and the crystals were ball-milled at 320 rpm for 48 h. After milling, the crystals were filtered and collected by size, first with an 8-μm pore size filter (ADVANTEC, K800A025A), followed by a 0.8-μm pore size filter (ADVANTEC, K080A025A).

Crystal size determination

The size and size distribution of the nanocrystals were determined by dynamic light scattering analysis using a DLS-8000 scatterometer (Otsuka, Japan). Crystals with a size of > 10 μm were directly observed using a polarised optical microscope (BX51, Olympus, Japan) to determine their crystal size.

S2-3. Photophysical properties

UV-visible absorption and steady-state photoluminescence spectra were recorded on a JASCO V-550 absorption spectrophotometer and a Hitachi F-7500 fluorescence spectrophotometer, respectively. Quantum yields of RTP were determined using a Quantaurus-QY absolute photoluminescence quantum yield spectrometer (C11347-01, Hamamatsu).

S2-4. Single-crystal X-ray structure analysis

The molecular structure and crystal packing structure were determined by single-crystal X-ray structural analysis. Single crystals of gold(I) complexes were obtained by slow evaporation from a mixed solvent system (dichloromethane/acetone). Each crystal was mounted on a glass fibre, and the omega scanning technique was used to collect the reflection data using a Bruker D8 goniometer with monochromatic Mo *K*α radiation (λ = 0.71075 Å) for DT5 and DT6, or a Rigaku automated four-circular-axis diffractometer AFC-5R with graphite monochromatised Cu *K*α radiation (λ = 1.54178 Å) for DT4. To investigate the actual crystal structure of the materials used, the measurements were performed at ambient temperature (296 – 300 K). For DT5 and DT6, the initial structure of each unit cell was determined using a direct method in APEX3. The structural models were refined using the full-matrix least squares method in SHELXL-2014/6.[6,7] All calculations were performed using the SHELXL program. For DT4, the initial structure in the unit cell was determined by a direct method using SIR92.[8] The structure model was refined by a full-matrix least-squares method using SHELXL97.[9] All calculations were performed using the crystallographic software package WinGX.[10] When the alkyl chains were disordered, the occupancy of the atoms was separated into two segments. The crystal data are summarised in Supplementary Table 1. The data for new compounds, DT4 and DT5, in Supplementary Table 1 have been indexed and are included in the Cambridge Crystallographic Centre (CCDC) database with the following reference numbers: CCDC 1973710 for DT4, and CCDC 1973709 for DT5. The indexed database contains additional supplementary crystallographic data for this paper and may be accessed without charge at http://www.ccdc.cam.ac.uk/conts/retrieving.html.

S2-5. Powder X-ray diffraction analysis of nanocrystals

The nanocrystals were collected from the suspension by filtration (filter pore size = 0.1 μm, ASONE, 025010MFPES). The residual nanocrystals on the filter were used directly for the measurement. The diffraction patterns of the nanocrystals on the filter were recorded on a powder X-ray diffractometer (Panalytical, X'Pert Pro MRD) with Cu *Kα*-1 Cu *Kα*-2 radiation ($\lambda_{K\alpha\text{-}1}$ = 1.540598 Å, $\lambda_{K\alpha\text{-}2}$ = 1.544426 Å, respectively. The ratio of *Kα*-1/*Kα*-2 = 1/1).

S2-6. Computational studies

Time-dependent density functional theory (TD-DFT) calculations were performed using the Gaussian 03 (revision E.01) program package, employing B3LYP hybrid functionals with SDD (for the Au atoms) and 6-311+G(d,p) basis sets for all other atoms.[11] Computation was performed for the dimer formed in the crystal, using the structural arrangement obtained by single-crystal X-ray crystallography. The vertical excitation energies and oscillator strengths were estimated for the 10 lowest transitions to excited singlets.

The association energy for dimer formation was estimated by the difference in total energy obtained by DFT calculations between the dimers in the single crystal and the dimer separated each molecule by a distance of 50 Å for the closest intermolecular Au–Au atoms.

S3. Supplementary Note 1

S3-1. Molecular structure and crystal packing structure

The molecular and crystal packing structures were determined using single-crystal X-ray structural analysis. The key crystallographic data are summarised in Supplementary Table 1.

Supplementary Table 1. Crystallographic data of complexes DT4–DT6 obtained at room temperature.

Complex	DT4	DT5	DT6
Radiation type	Cu Kα	Mo Kα	Mo Kα
Wavelength (Å)	1.54178	0.71073	0.71073
Empirical formula	$C_{27}H_{45}Au_3N_6$	$C_{30}H_{51}Au_3N_6$	$C_{33}H_{57}Au_3N_6$
Formula weight	1044.59	1086.67	1128.75
Temperature (K)	296	300	296
Crystal habit	plate	plate	plate
Crystal colour	colourless	colourless	colourless
Crystal size (mm)	0.55 x 0.11 x 0.02	0.47 x 0.28 x 0.06	0.74 x 0.08 x 0.04
Crystal system	Triclinic	Monoclinic	Monoclinic
Space group	*P -1*	*P2(1)/c*	*P 21/n*
a (Å)	5.8889(5)	15.9459(5)	17.8775(6)
b (Å)	13.9282(13)	22.9415(6)	8.2352(2)
c (Å)	20.468(2)	9.5469(3)	26.2483(8)
α (deg)	107.558(7)	90.00	90.00
β (deg)	95.891(10)	92.9990(10)	91.0320(10)
γ (deg)	98.396(8)	90.00	90.00
V (Å^3)	1564.2(3)	3487.69(18)	3863.8(2)
Z	2	4	4
$R\,[F^2 > 2\sigma(F^2)]$	0.0602	0.0390	0.0413
$wR\,(F^2)$	0.1832	0.1058	0.1657

Supplementary Table 2. Selected geometrical parameters obtained by crystallography, and intermolecular interactions and association energies estimated for dimer formation by DFT calculations for the complexes in the crystal.

	Au–Au		Au–pyrazolyl			Association energy for dimer formation[d] (kJ/mol)
	d[a] (Å)	interaction	*d*[b] (Å)	*θ*[c] (°)	interaction	
DT4	3.657	yes	3.843	27	no	20.8
DT5	4.369	no	3.708<br>3.740	18<br>19	yes<br>yes	19.1
DT6	3.400<br>3.427	yes<br>yes	3.584<br>3.638	15<br>15	yes<br>yes	34.5

[a] Interatomic distance between two Au atoms in neighbouring molecules. [b] Distance between the Au atom and the centroid of the pyrazolyl ring in a neighbouring molecule. [c] Angle between the vector normal to the pyrazolyl ring and the vector passing through the centroid to the Au atom. [d] Estimated by DFT calculation using B3LYP/SDD (for Au atoms) and 6-311+G(d,p) (for other atoms).

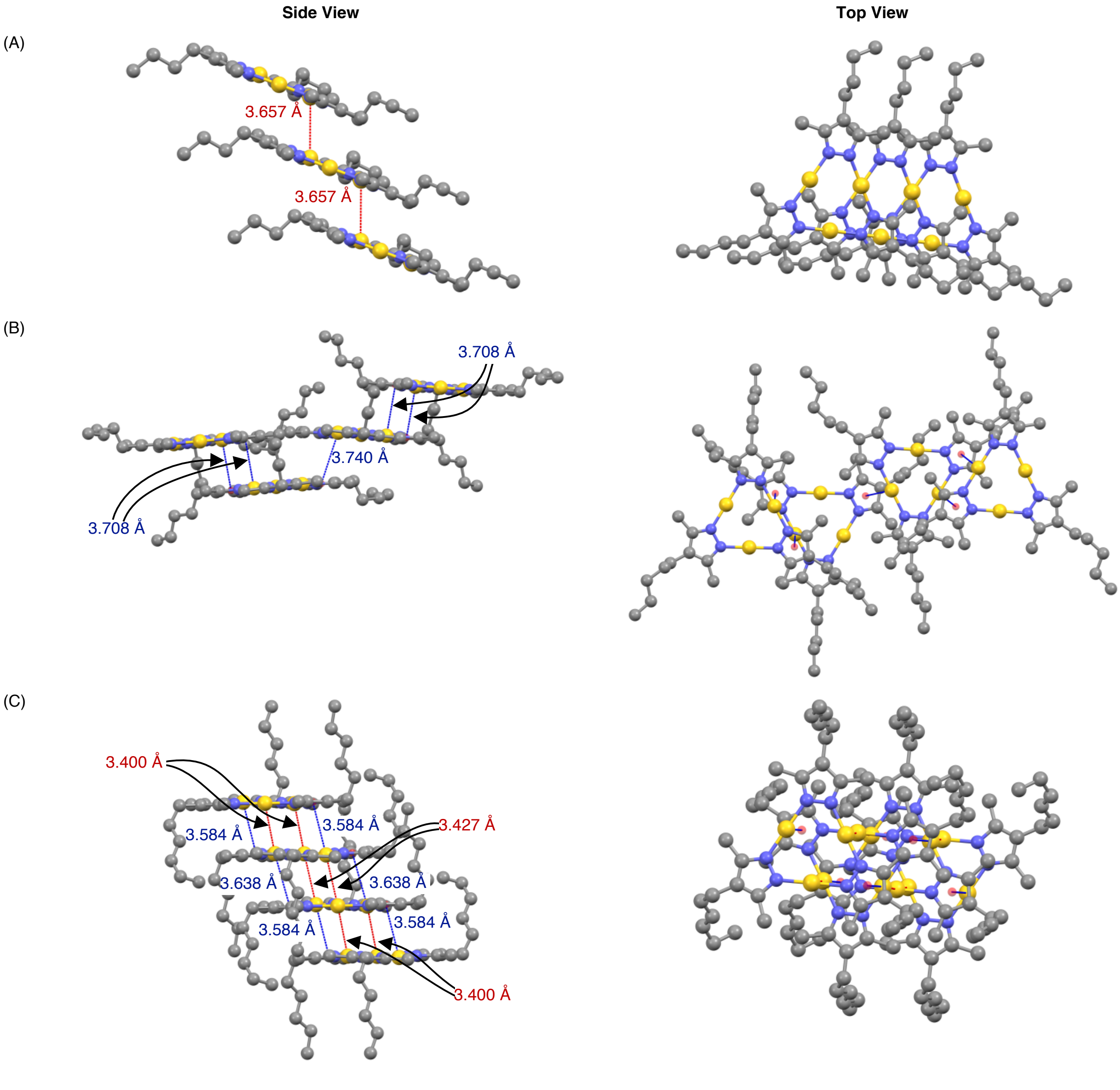

Supplementary Figure 3. Crystal structures of DT*n* at room temperature. Supramolecular polymers formed in bulk crystals are shown: (A) DT4, (B) DT5, and (C) DT6. For clarity, the H atoms are omitted. Atom colour legend: grey, C; purple, N; yellow, Au; red, the centroid of pyrazole ring. Intermolecular Au–Au and Au–π interactions are indicated with red and blue lines, respectively.

S3-2. Characterisation of micro- and nanocrystals

Size distribution

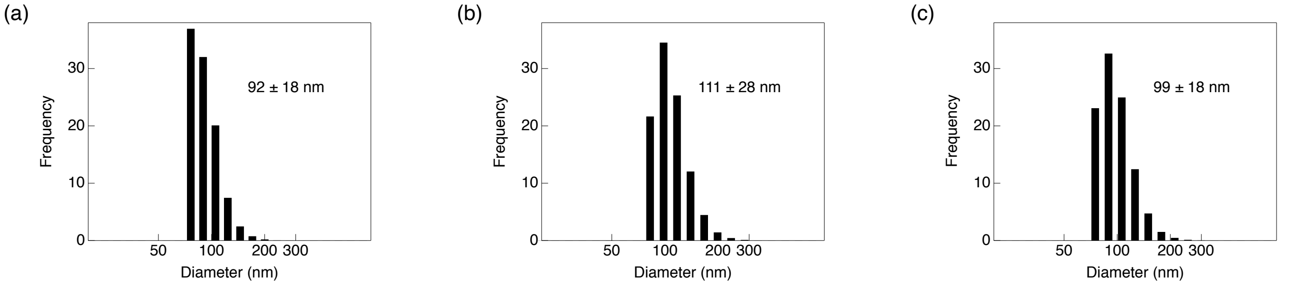

Supplementary Figure 4. Size distributions of the Au-complex nanocrystals prepared by reprecipitation from THF/H_2O: (a) DT4; (b) DT5; (c) DT6.

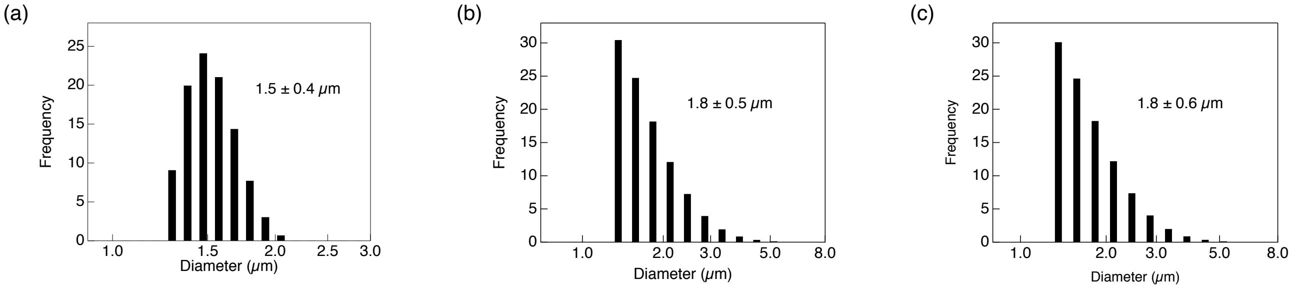

Supplementary Figure 5. Size distributions of the Au-complex microcrystals immediately after preparation by reprecipitation from $CHCl_3$/methanol: (a) DT4; (b) DT5; (c) DT6.

S3-3. Photophysical properties

Absorption and luminescence behaviour

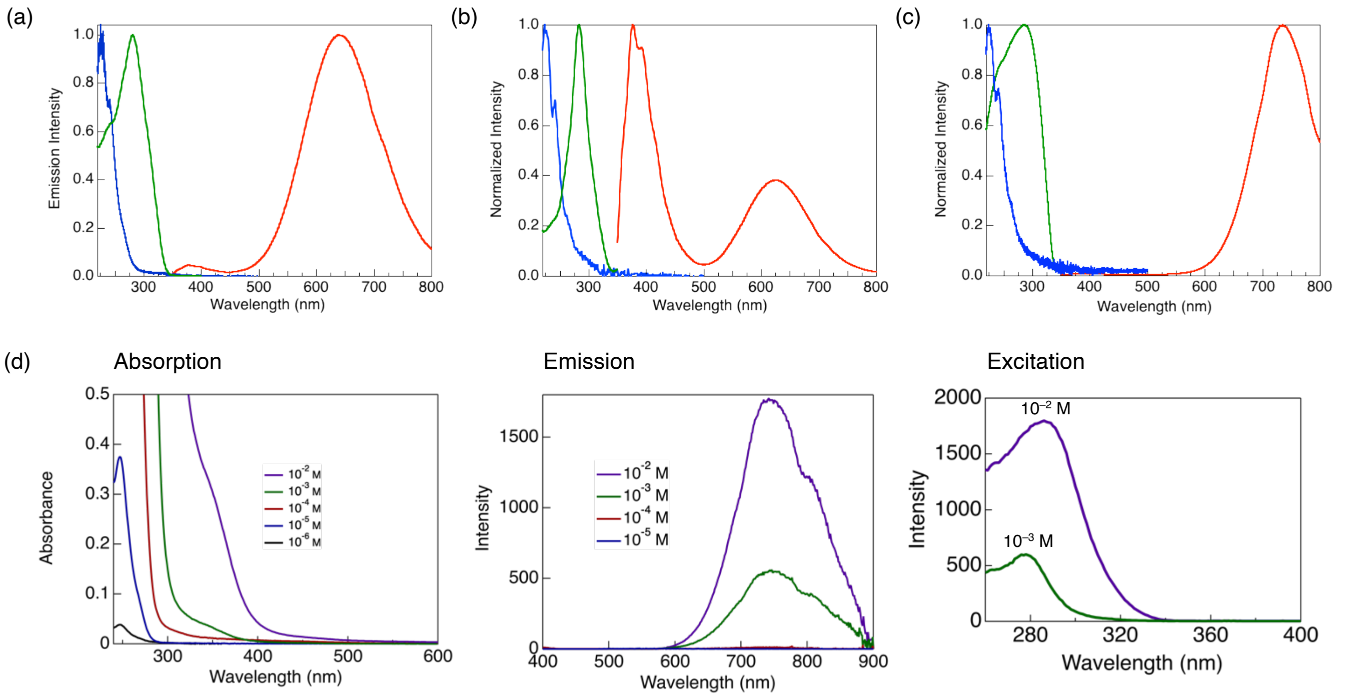

Supplementary Figure 6. (a–c) Absorption spectra in dilute hexane solution (10^{-5} mol L^{-1}, blue), photoluminescence spectra in the crystal (red), and excitation spectra in the crystal (green) of DT4–DT6: (a) DT4 (λ_{ex} = 280 nm for luminescence, λ_{em} = 638 nm for excitation); (b) DT5 (λ_{ex} = 280 nm for luminescence, λ_{em} = 377 nm for excitation); (c) DT6 (λ_{ex} = 280 nm for luminescence, λ_{em} = 733 nm for excitation). (d) Concentration dependence of absorption, luminescence, and excitation spectra of DT4 in CH_2Cl_2 solutions (λ_{ex} = 280 nm for luminescence, λ_{em} = 740 nm for excitation): concentration of the solutions is indicated in the figures.

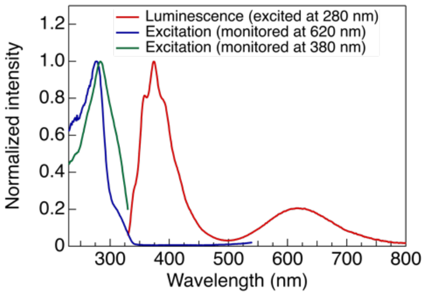

Supplementary Figure 7. Luminescence and excitation spectra of DT5 bulk crystal observed at room temperature. (red), luminescence (λ_{ex} = 280 nm); (blue), excitation (λ_{em} = 620 nm); (green), excitation (λ_{em} = 380 nm).

AIE properties

To confirm the AIE activity of the complexes, luminescence spectra were measured in solvent mixtures of good and poor solvents; where good and poor refer to whether the crystals were soluble or insoluble in the solvents, respectively. Here, CH_2Cl_2 and methanol were used as the good and poor solvents, respectively. In a neat CH_2Cl_2 solution, the complexes exhibited no luminescence; however, the addition of MeOH to the solution with a volume fraction of >80% induced the aggregation of molecules and significantly enhanced luminescence.

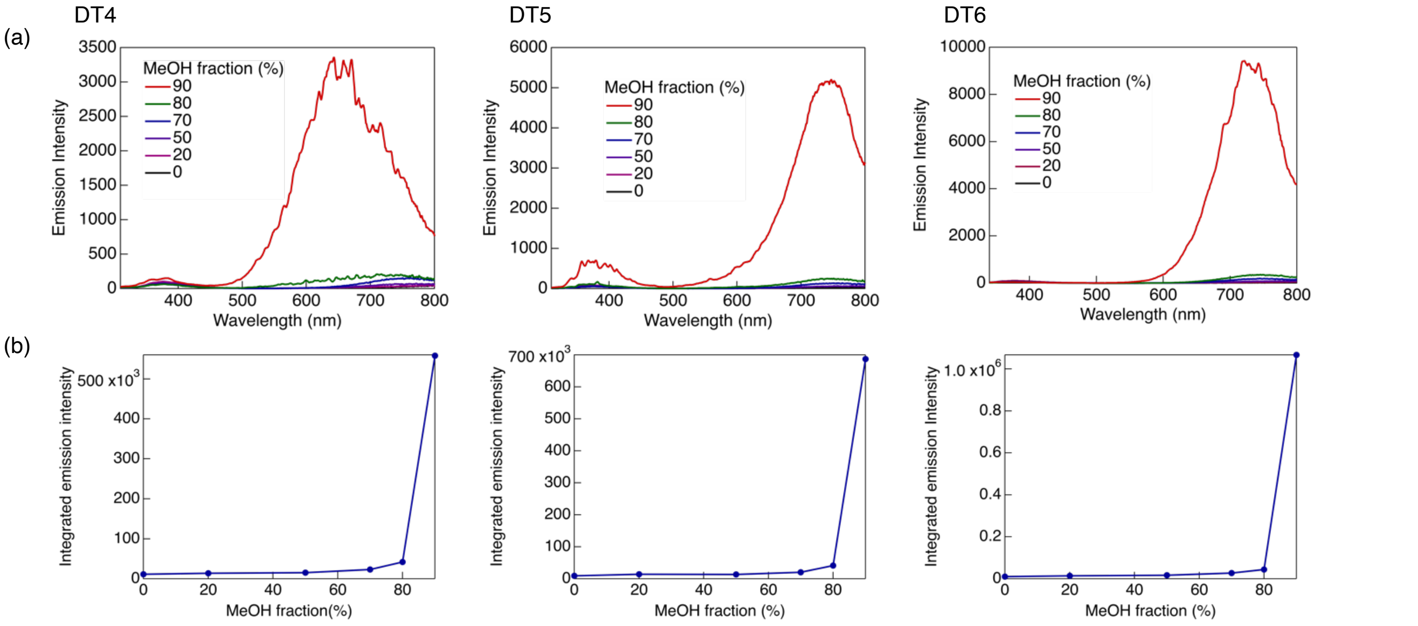

Supplementary Figure 8. (a) Photoluminescence spectra of DT*n* in CH_2Cl_2/methanol mixtures with varying methanol concentrations ([DT*n*] = 2.0×10^{-5} mol L^{-1}, λ_{ex} = 250 nm) under ambient conditions. The methanol fractions (vol%) in the solvent are indicated in the figure. (b) Integrated emission intensity of DT*n* in the CH_2Cl_2/methanol mixture as a function of methanol fraction.

Decay profile in the crystal at room temperature

The decay profiles of complexes in the crystal were recorded (Supplementary Figure 9–S11), and the results are summarized in Table 1 in the main text.

As shown in Fig 1c, two luminescent bands (major and minor bands) appeared in the spectrum of DT4 and DT5 bulk crystals: the major band was a low energy band in the DT4 bulk crystal, and it was a high energy band in the DT5 bulk crystal. In both DT4 and DT5 bulk crystals, the high energy band showed bi-exponential decay profiles. In addition, the rise and decay were observed at the low energy bands. In both complexes, the rise time at the low energy band was roughly the same as the fast decay at the high energy band. The results suggest that the energy transfer or structural relaxation of the crystal occurred in the excited state.

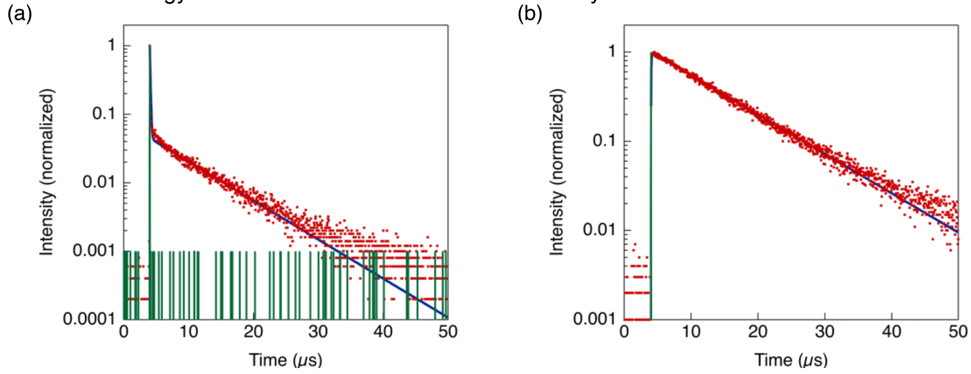

Supplementary Figure 9. Decay profiles for room-temperature phosphorescence in bulk crystals of DT4 monitored 390 nm (a, minor band) and at 615 nm (b, major band): red, observed luminescence decay; blue, fitting curve; green, instrument response function.

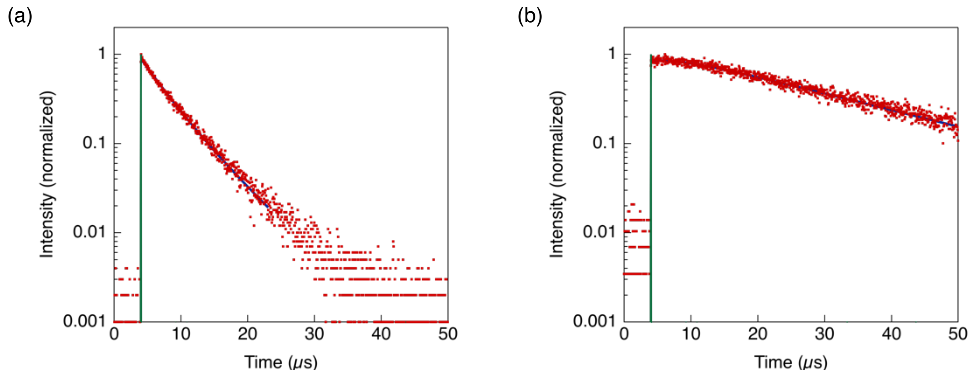

Supplementary Figure 10. Decay profiles for room-temperature phosphorescence in bulk crystals of DT5 monitored at 405 nm (a, major band) and 605 nm (b, minor band): red, observed luminescence decay; blue, fitting curve; green, instrument response function.

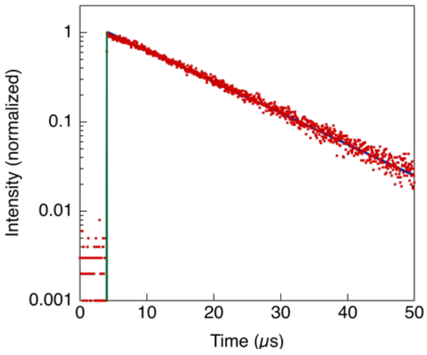

Supplementary Figure 11. Decay profiles for room-temperature phosphorescence in bulk crystals of DT6 monitored at 700 nm: red, observed luminescence decay; blue, fitting curve; green, instrument response function.

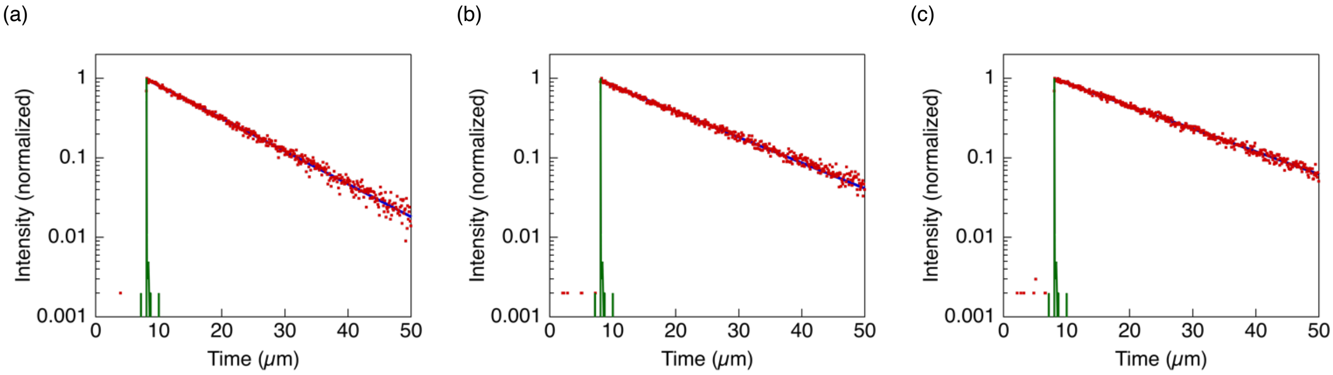

Supplementary Figure 12. Decay profiles for room-temperature phosphorescence in nanocrystals of (a) DT4, (b) DT5, and (c) DT6 on filters monitored at 700 nm: red, observed luminescence decay; blue, fitting curve; green, instrument response function.

S3-4. DFT calculations

The molecular orbitals, transition energies, and oscillator strengths (*f*) of the complexes were calculated using time-dependent density functional theory (TD-DFT). The calculations were performed for dimers formed in the crystals using the arrangements shown in Figure 2.

For comparison, the TD-DFT calculation was performed also for the monomer of the Au complex. Here, to reduce the calculation load, complex **DT1** was employed as a model compound for the calculation (Supplementary Figure 16). For the monomer, we employed the same hybrid functionals and basis sets as the dimer for both geometry optimization and vertical excitation energy calculation. Stationary points were characterized by frequency calculations to ensure the correct number of imaginary frequencies, and we confirmed that the minimum energy structures had no imaginary frequencies. The vertical excitation energies and oscillator strengths were estimated for the 10 lowest transitions for the optimized equilibrium geometries using TD-DFT.

The TD-DFT calculations for the dimers suggested that electronic transitions from the ground state (S_0) to the singlet excited state (S_n) are located in the UV region between 254 and 262 nm (Supplementary Table 3). On the other hand, in case of the monomer, the S_0–S_n transitions are located at 244 nm, and this calculated result was roughly consistent with the absorption spectra of the complex

in dilute solution (Supplementary Figure 6). Thus, it can be concluded that 10–20 nm shift of the absorption wavelength was induced by dimer formation. Furthermore, the calculations suggested that an intramolecular LMMCT transition occurred in the monomer (Supplementary Figure 16).

Supplementary Table 3. Transition, oscillator strength (*f*), and excitation wavelength (λ) for S_0–S_n excitation calculated for the dimer of the complexes.

Complex	Transition			*f*	λ (nm)
DT4	HOMO	→	LUMO+4	0.0105	254.41
	HOMO–1	→	LUMO+4	0.0313	252.59
DT5	HOMO–1	→	LUMO+1	0.0220	262.07
	HOMO–4	→	LUMO	0.0342	261.08
DT6[2]	HOMO	→	LUMO+2	0.0292	255.46

Supplementary Table 4. Excitation wavelength (λ) for the lowest singlet-to-triplet transition calculated for the dimer of the complexes.

Complex	λ (nm)
DT4	292.63
DT5	297.18
DT6	310.63

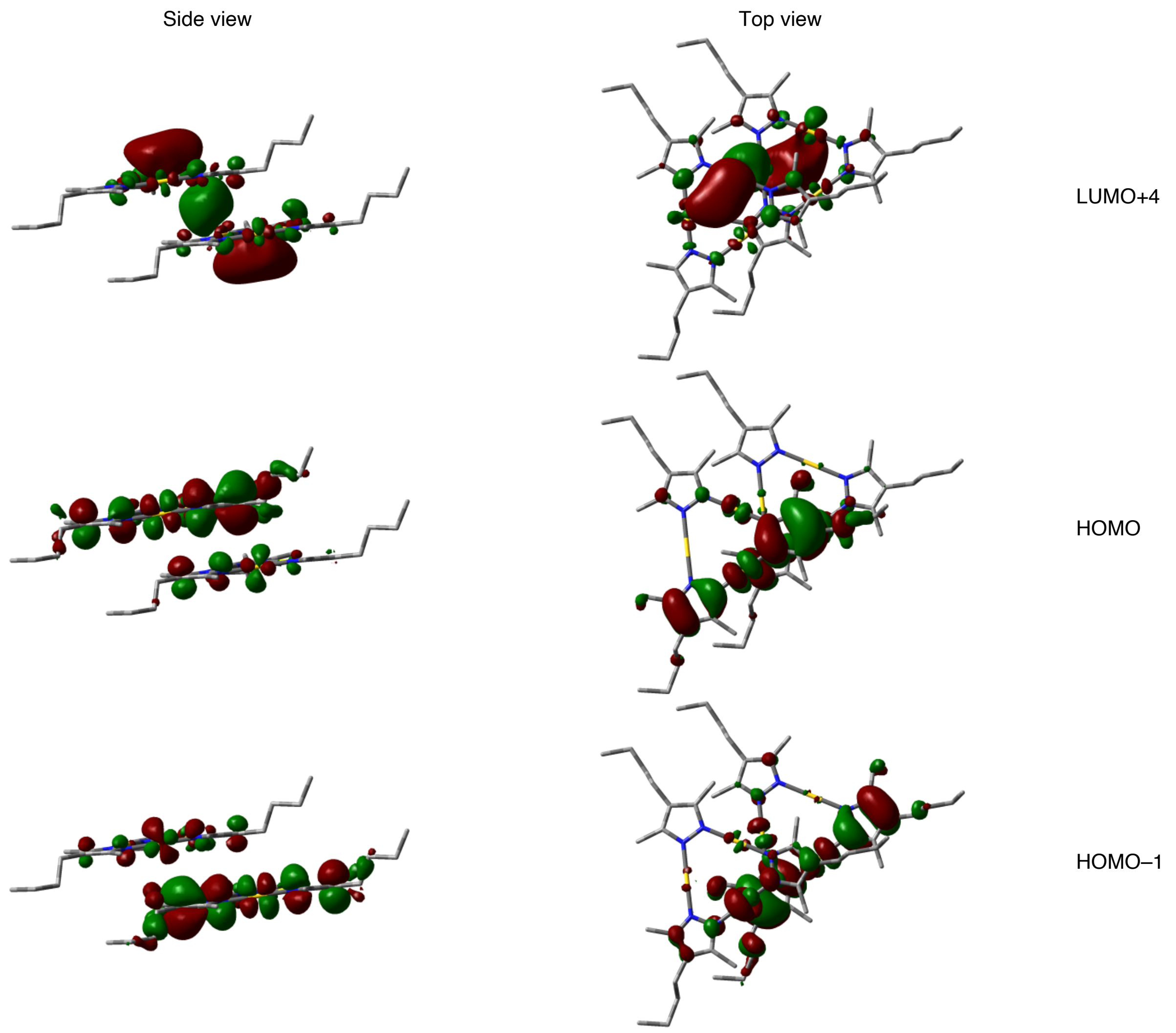

Supplementary Figure 13. Molecular orbitals. of the DT4 dimer obtained by DFT calculations. For clarity, the H atoms are omitted. Atom colour legend: grey, C; blue, N; yellow, Au.

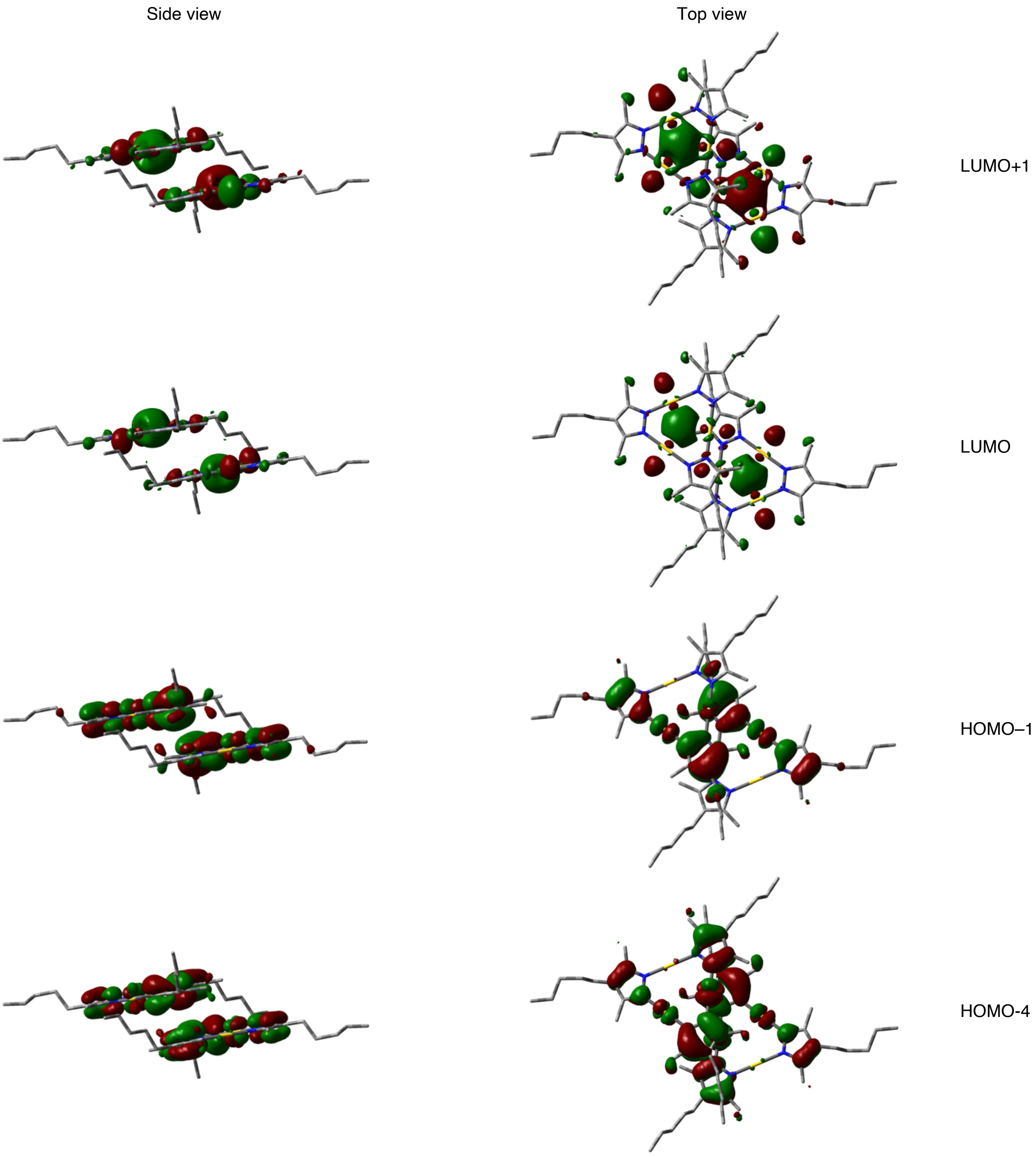

Supplementary Figure 14. Molecular orbitals. of the DT5 dimer obtained by DFT calculations. For clarity, the H atoms are omitted. Atom colour legend: grey, C; blue, N; yellow, Au.

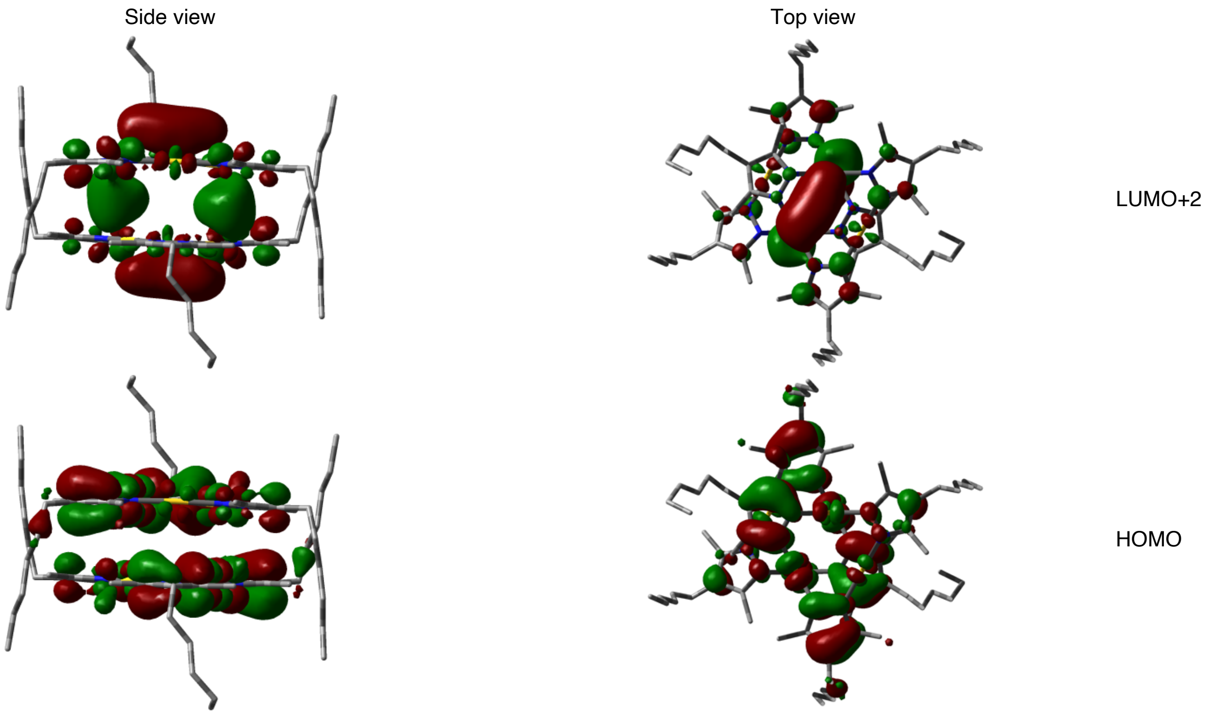

Supplementary Figure 15. Molecular orbitals. of the DT6 dimer obtained by DFT calculations.[5] For clarity, the H atoms are omitted. Atom colour legend: grey, C; blue, N; yellow, Au.

Supplementary Table 5. Transition, oscillator strength (*f*), and excitation wavelength (*λ*) calculated for the monomer of the complex **DT1**.

Complex	Transition			*f*	*λ* (nm)
**DT1**	HOMO	→	LUMO+3	0.0629	244.12
	HOMO–1	→	LUMO+3	0.0504	243.57

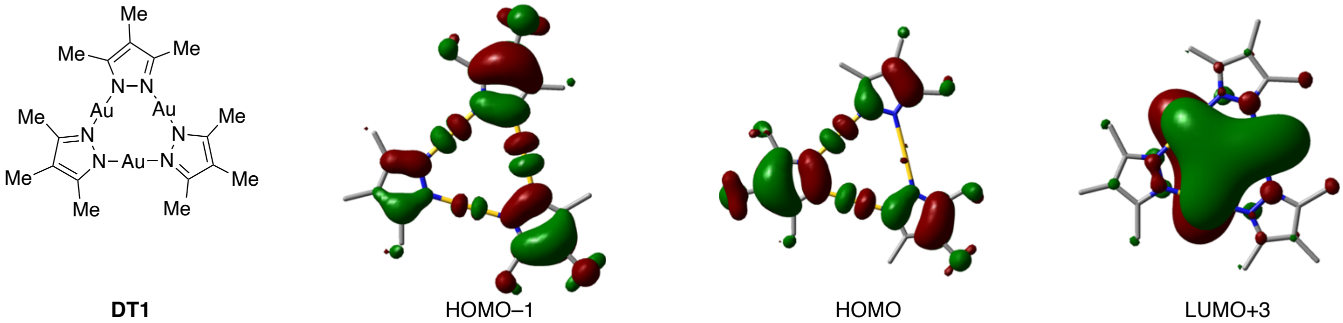

Supplementary Figure 16. DTF calculation for the monomer of the Au complex: molecular structure of **DT1** calculated as a model compound, and molecular orbitals. of the **DT1** monomer. For clarity, the H atoms are omitted. Atom colour legend: grey, C; blue, N; yellow, Au.

S3-5. Effects of solvent on crystal structure and luminescence behaviour

Luminescence behaviour of microcrystals.

Photoluminescence spectra of microcrystals prepared by reprecipitation from $CHCl_3$ (5.0 mmol L^{-1}, 500 μL)/MeOH (10 mL) are shown in Supplementary Figure 17. The microcrystals were collected by filtration immediately after reprecipitation to prevent crystal growth, and their luminescence spectra were measured whilst they were on the filters. Similar to the nanocrystals obtained from THF/water, all the complexes exhibited red luminescence immediately after reprecipitation, indicating the absence of solvent effects on the luminescence behaviour of nano-/microcrystals. During the crystal growth process, the luminescence spectra and colours of DT4 and DT5 changed to those of the bulk crystal. After two days, the luminescence spectra were identical to those of the bulk crystals. From these experiments, we can conclude that complexes DT4 and DT5 underwent crystalline polymorphism, and that a phase

transition between polymorphs occurred during crystal growth. In contrast, the luminescence spectrum of DT6 immediately following reprecipitation resembled the spectrum of bulk crystals and remained unchanged, even after the crystals grew to a size of 50 μm.

The detailed time evolution of the photoluminescence spectra of the microcrystal is shown in Supplementary Figure 18 for DT4 as a representative example. The measurement was performed at 18 °C. The crystal growth rate is sensitive to the measurement temperature. At this temperature, the suspension reached the equilibrium almost at 10 h, and the same luminescence spectrum with the bulk crystal was obtained. In addition, in first 6 h, no significant change in the luminescence spectrum was observed; however, the sharp change was observed between 6 and 10 h. In the suspension, the microcrystals grew gradually through Ostwald ripening. When the crystal radius reaches the crossing point of the energy curves of the polymorphs shown in Figure 4f, a metastable-to-stable crystal phase transition takes place. Thus, it is reasonable that the sharp spectral change was observed at the specific time between 6 and 10 h, and this sharp change support our conclusion that the luminescence colour change in the present complexes occurred by the crystal–crystal phase transition induced by the crystal growth.

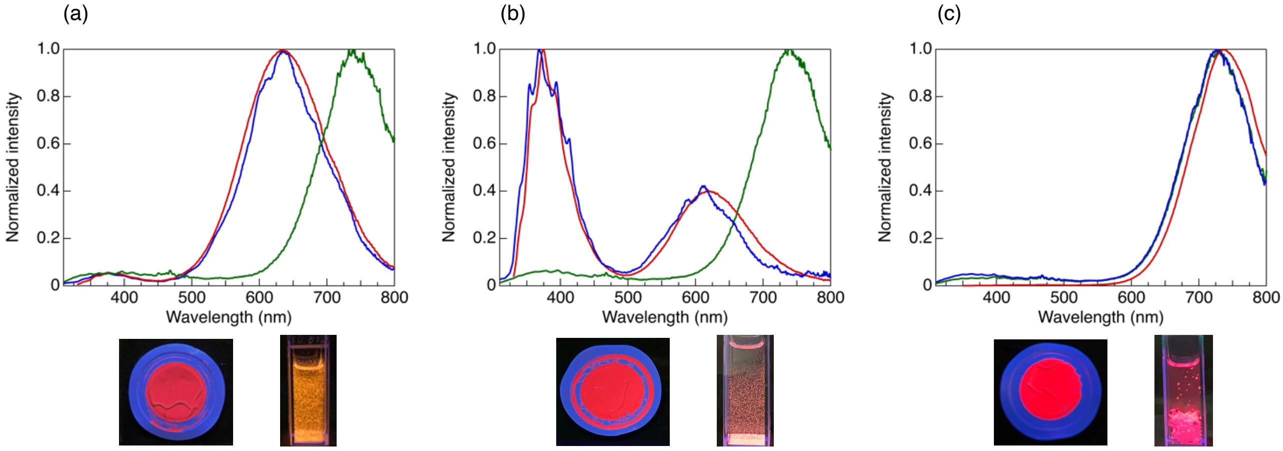

Supplementary Figure 17. Luminescence behaviour of DT*n* crystals: (a) DT4; (b) DT5; (c) DT6. (top) Photoluminescence spectra of DT*n* crystals (excitation at 280 nm). Green: microcrystals immediately after reprecipitation from $CHCl_3$ (5.0 mmol L^{-1}, 500 μL)/MeOH (10 mL) solution measured on filters with a pore size of 0.1 μm; blue: reprecipitated crystals grown for 48 h in a methanol suspension; red: bulk crystals prepared by conventional recrystallisation. (bottom) Photographs of crystals taken under irradiation at 254 nm. Left: immediately after reprecipitation; right: crystals grown for 48 h in a methanol suspension.

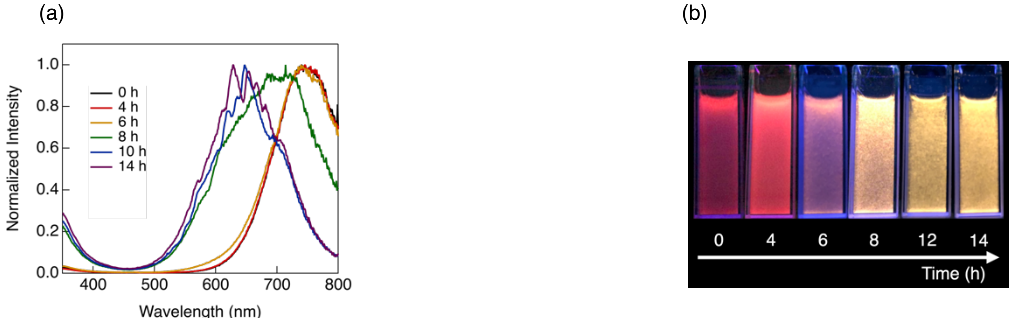

Supplementary Figure 18. (a) Detailed time evolution of the photoluminescence spectra (excitation at 280 nm) of DT4 microcrystals in a methanol suspension prepared by precipitation from $CHCl_3$/MeOH (500 μL/10 mL). The measurement was performed at room temperature (~18 °C). (b) Photographs of DT4 crystal suspensions taken under irradiation at 254 nm. The suspension was kept in the dark at room temperature (~18 °C), and the photographs were taken at time indicated in the figure after sample preparation.

Effects of solvent on the luminescence behaviour of the bulk crystal

Bulk crystals of the complexes were prepared from various solvent systems. Well-purified complexes (typically ~150 mg) were dissolved in 30 mL of a good solvent, and then 30 mL of a poor solvent was gently poured into the resultant solution. The solution was allowed to stand for at least two days to obtain bulk crystals with sizes >100 μm. Combinations of CH_2Cl_2/acetone or THF/water were used as good/poor solvents. Both DT5 bulk crystals prepared from the two different solvent systems exhibited the same luminescence spectra with a major luminescence band at 380 nm, as shown in Supplementary Figure 19.

When the nanocrystals were prepared from the THF/water solvent system, the DT5 complex exhibited red luminescence (λ_{max}^{lum} = 736 nm, Figure 3a in the main text). Although the crystals were prepared from the same solvent system, the luminescence colours were different between the nanocrystals and bulk crystals; this implies that the luminescence colour in DT4 and DT5 is highly dependent on the structure of molecular aggregates, which are altered by the crystal size. The results shown in Figs. S17–19 and Figure 3 clearly indicate that the luminescence behaviour of the present Au complexes depends only on the crystal-size-dependent aggregated structures, and that the solvent effects can be ruled out of the luminescence behaviour.

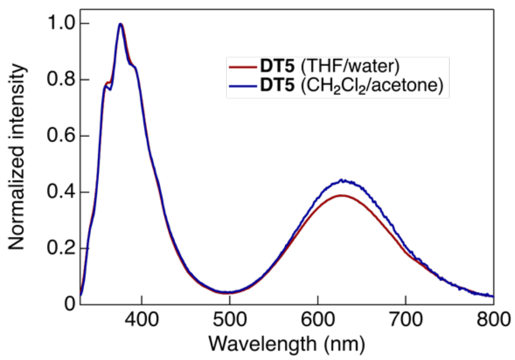

Supplementary Figure 19. Photoluminescence spectra of DT5 bulk crystals prepared by recrystallisation from THF/water (red) and CH_2Cl_2/acetone (blue).

Powder X-ray diffraction

As stated in the main text, the luminescence results for the nanocrystals suggest that similar luminescent species with the same intermolecular interactions are formed by all the complexes present in the nanocrystals. Thus, the crystal structures of complexes DT4 and DT5 should differ between the nanocrystals and the bulk crystals. To confirm this assumption, as stated in the main text, we performed powder X-ray diffraction (XRD) analysis of the nanocrystals prepared by reprecipitation from THF/water (Figure 3b). Here, we also observed XRD patterns for the microcrystals prepared from a different solvent system ($CHCl_3$ (5.0 mmol L^{-1}, 500 μL)/MeOH (10 mL)) to evaluate the solvent effects. As mentioned in the main text, the microcrystals grew in the methanol suspension through Ostwald ripening; therefore, the XRD patterns were observed immediately after reprecipitation and after crystal growth for two days (Supplementary Figure 20). As shown in Supplementary Figure 17, immediately after reprecipitation, the microcrystals showing red luminescence were obtained from all the complexes. However, after 2 days of growth, crystals with sizes of 50–100-μm exhibiting the same morphology as the corresponding bulk crystals were obtained (Supplementary Figure 20), and they exhibited luminescence spectra akin to those of the bulk crystals (Supplementary Figure 18).

As shown in Supplementary Figure 20, immediately after reprecipitation, the microcrystals of the DT4 and DT5 complexes exhibited crystal structures distinct from those of the bulk crystals prepared by the common recrystallisation method from CH_2Cl_2/acetone. In contrast, after 2-days of growth, the reprecipitated crystals gave rise to the same XRD pattern as the bulk crystals. These results indicate that a phase transition between polymorphs occurred during the crystal growth process. In addition, as shown in Supplementary Figure 20, the XRD patterns of the microcrystals were identical to those of the nanocrystals obtained from the THF/water solvent system. Thus, solvent effects can be ruled out as governing factors for crystal structure in the present complexes. For DT6, however, no change in the XRD pattern was observed during crystal growth, and all crystals exhibited the same XRD pattern, resembling that of the bulk crystals.

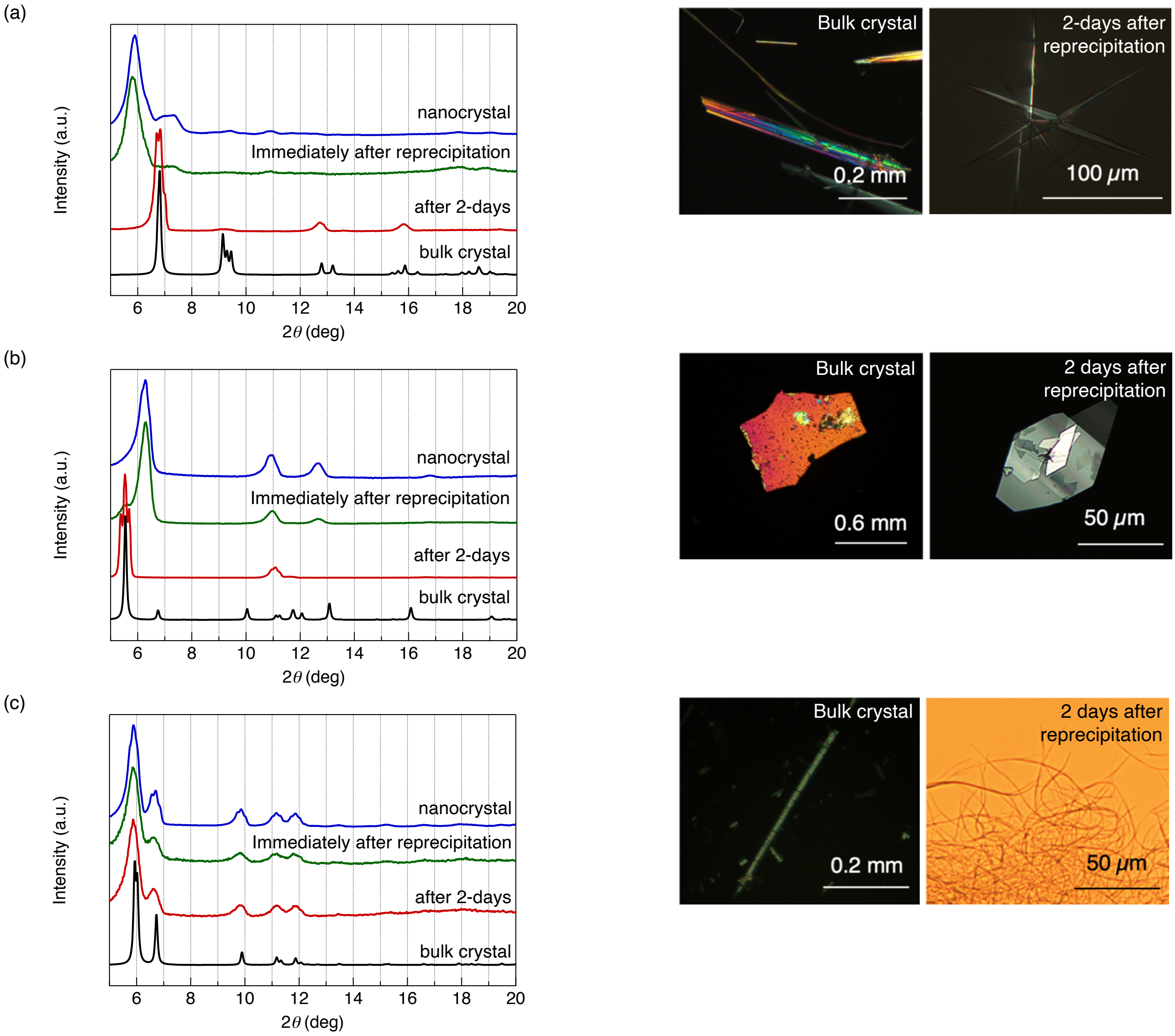

Supplementary Figure 20. (left) Powder XRD patterns of DT*n* crystals: (a) DT4; (b) DT5; (c) DT6. Blue: nanocrystal prepared from THF/water, green: microcrystal immediately after reprecipitation from $CHCl_3$/MeOH, red: 2-days after the reprecipitation, black: XRD patterns of bulk crystals simulated from the single-crystal X-ray structures. (right) Photographs of the recrystallised bulk crystals and reprecipitated crystals after 2-days of growth in MeOH suspension.

Conclusion of solvent effects on crystal structure and luminescence behaviour

In this section, the effects of solvent on the crystal structure and luminescence behaviour were investigated. In bulk crystals and nano-/microcrystals of complexes DT4 and DT5, the crystal structure and luminescence behaviour did not depend on the solvent system used for crystallisation. However, their crystal structure and luminescence behaviour strongly depended on the size of the crystals. These results support our conclusion stated in the main text that certain materials exhibit contrasting behaviour when they are made smaller (<10 μm in the case of DT4 and DT5). The behaviour of materials of reduced size may not be the same as that of the corresponding bulk crystals; thus, attention should be paid to the size of the materials when considering their characteristics.

S3-6. Mechanical-force effects on crystal structure and luminescence behaviour

In common luminescent mechanochromic materials, changes in the crystal structure and luminescence behaviour can be induced by hand grinding with a mortar and pestle. According to the literature, it is very challenging to prepare particles with sizes of less than 10 μm using this method.[13] In our study, the bulk crystals were pulverised by ball milling at 340 rpm for 48 h to obtain pulverised microcrystals with sizes of 100 nm to 10 μm. Considering that luminescent mechanochromism can be induced by hand grinding of common mechanochromic materials, the ball-milling process used to obtain the microcrystals in this study should have supplied sufficient mechanical energy to induce a luminescence colour change. Nevertheless, crystals with a size of ~8 μm (**Residual 1**) exhibited the same luminescence behaviour as the original bulk crystals. These results suggest that the mechanical force effects can be disregarded in DT*n* crystal luminescence behaviour. Thus, we conclude that the observed colour changes were as a result of differences in crystal sizes.

Supplementary References

1. R. Uson, A. Laguna, M. Laguna, D. A. Briggs, H. H. Murray, J. P. Fackler Jr. *Inorg. Synth.* **2007**, *26*, 85–91;
2. J. Barberá, A. Elduque, R. Giménez, L. A. Oro, J. L. Serrano, *Angew. Chem.*, *Int. Ed. Engl.* **1996**, *35*, 2832–2835;
3. S. J. Kim, S. H. Kang, K. M. Park, H. Kim, W. C. Zin, M. G. Choi, K. Kim, *Chem. Mater.* **1998**, *10*, 1889–1893;
4. J. Cored, O. Crespo, J. L. Serrano, A. Elduque, R. Giménez, *Inorg. Chem.* **2018**, *57*, 12632–12640.
5. O. Tsutsumi, M. Tamaru, H. Nakasato, S. Shimai, S. Panthai, Y. Kuroda, K. Yamaguchi, K. Fujisawa, K. Hisano, *Molecules* **2019**, *24*, 4606.
6. G. M. Sheldrick, *SHELXS-2014, Program for Crystal Structure Solution*, University of Göttingen, 2014;
7. G. M. Sheldrick, *A Short History of SHELX, Vol. 64, Acta Crystallogr., Sect. A*: *Found. Crystallogr.* **2008**, *64*, 112–122.
8. A. Altomare, G. Cascarano, C. Giacovazzo, A. Guagliardi, *J. Appl. Crystallogr.* **1993**, *26*, 343–350.
9. G. Sheldrick, *Acta Crystallographica Section A*, **2008**, *64*, 112-122.
10. L. J. Farrugia, *J. Appl. Crystallogr.*, **1999**, *32*, 837-838.
11. M. J. Frisch, et al., Gaussian 03, Revision E.01, Gaussian, Inc., Wallingford, CT, USA **2004**.
12. M. A. Omary, et al. Metal effect on the supramolecular structure, photophysics, and acid−base character of trinuclear pyrazolato coinage metal complexes. *Inorg. Chem.* **44,** 8200–8210 (2005)
13. D. L. Bish, M. Plötze in *Advances in the Characterization of Industrial Minerals* (Ed.: G. E. Christidis), Mineralogical Society of Great Britain & Ireland, London, 2010, pp. 52–56.
