# Supplementary material for: Observation of crystallisation dynamics by crystal-structure-sensitive room-temperature phosphorescence from Au(I) complexes
Source: Commun Chem. 2020 Oct 14;3:139. doi: 10.1038/s42004-020-00382-1 (PMC9814381; doi:10.1038/s42004-020-00382-1)
Supplement: Supplementary file 2 — Description of Additional Supplementary Files [file 42004_2020_382_MOESM2_ESM.pdf]

Description of Additional Supplementary Files

File Name: Supplementary Data 1
Description: CIF for DT4

File Name: Supplementary Data 2
Description: CIF for DT5

File Name: Supplementary Data 3
Description: CIF for DT6
